# To the Editors of the Medical and Physical Journal

**Published:** 1809-05

**Authors:** William Blair

**Affiliations:** Great Russel Street, Bloomsbury Square


### *To the Editors of the Medical and Physical Journal.*

GENTLEMEN,

IN the Medical and Physical Journal for February last, at p. 122, are some very harsh and personal reflections, made by Mr. Davies, on a "Deputation" of the Jennerian Society; as if they were "men so blinded by vulgar prejudices, and so *wilfully* deaf to the voice of conviction," that they can never "admit the existence of any thing like failure, in a practice which they have made it their peculiar business to hold out as infallible."

The writer of these remarks has complimented himself on his own impartiality and candour! It is therefore presumed, that he will not refuse to give an explicit reply to the following questions.

1. Am I included in the censure conveyed by the above quotation?

2. If I am, what are the grounds on which he ventures to insinuate such a charge of obliquity in my moral and medical character?

3. Has he read all I have published on Vaccination, and especially my case of failure recorded in Dr. Willan's Treatise upon this subject?

WILLIAM BLAIR.

*Great Russel Street, Bloomsbury Square, April* 25, 1809.

